# The Potential Benefits of Exercise in Managing Inflammatory Bowel Disease: A Systematic Review

**DOI:** 10.7759/cureus.68948

**Published:** 2024-09-08

**Authors:** Ghadeer Sabir, Hala A Abdelhady, Adoum Oumar Abakar, Ravindra Reddy Gangavarapu, Sayed A Mahmud, Anura Manandhar, Iana Malasevskaia

**Affiliations:** 1 Internal Medicine, California Institute of Behavioral Neurosciences & Psychology, Fairfield, USA; 2 Internal Medicine Clinical Research, California Institute of Behavioral Neurosciences & Psychology, Fairfield, USA; 3 Internal Medicine, Universidad de Ciencias Medicas de La Habana, Havana, CUB; 4 Medical Research, California Institute of Behavioral Neurosciences & Psychology, Fairfield, USA; 5 Research and Development, California Institute of Behavioral Neurosciences & Psychology, Fairfield, USA; 6 Obstetrics and Gynecology, Private Clinic "Yana Alexandr", Sana'a, YEM

**Keywords:** crohn's disease, disease progression, exercise, inflammatory bowel disease (ibd), physical activity, treatment, ulcerative colitis

## Abstract

Adults with inflammatory bowel disease (IBD) experience a significant decline in quality of life due to various symptoms. Exercise has emerged as a potential therapeutic approach to improve IBD management, but its effectiveness requires further investigation. This systematic review, adhering to PRISMA 2020 guidelines, explored the effects of exercise on IBD progression and its potential as a treatment in adults. A comprehensive search strategy was conducted across three databases and two registries from May 12, 2024, to May 22, 2024. Methodological rigor and potential bias were minimized through quality assessment using the Cochrane risk-of-bias tool 2 (RoB 2) for randomized controlled trials (RCTs), the Newcastle-Ottawa Scale (NOS) for cohort studies, and the Joanna Briggs Institute (JBI) critical appraisal checklist for studies evaluating the effectiveness of non-randomized interventions. This process yielded 12 high-quality studies for analysis. The review identified positive evidence from both RCTs and observational studies. Exercise interventions demonstrated improvements in cardiorespiratory fitness, disease activity, quality of life, and mental health in adults with IBD. Studies explored various modalities, including aerobic exercise, resistance training, and mind-body interventions. However, further research is needed to optimize exercise prescription and elucidate the underlying mechanisms of action. This review strengthens the evidence for exercise as a beneficial intervention for IBD patients. Personalized exercise programs based on individual needs hold promise for improved IBD management and patient outcomes. However, limitations exist due to study design variations and the need for long-term follow-up studies.

## Introduction and background

Inflammatory bowel disease (IBD) is a chronic inflammatory condition affecting the gastrointestinal tract. Crohn's disease and ulcerative colitis are the most common forms, with Crohn's primarily affecting the small intestine and ulcerative colitis affecting the large intestine [1]. These conditions significantly impact the quality of life for millions worldwide, causing symptoms such as abdominal pain, diarrhea, fatigue, and weight loss [1]. Additionally, up to 60% of IBD patients experience extra-intestinal symptoms, often musculoskeletal issues such as osteoporosis or muscle degeneration, further hindering their well-being [2].

The global prevalence of IBD is concerning, with an estimated 84.3 per 100,000 people affected in 2017 [3]. In North America alone, millions are diagnosed, with three million American adults in 2015, 725 per 100,000 in Canada by 2018, and estimates of 2.5 to three million in Europe [4,5]. While sociodemographic factors such as age, race, education, and poverty influence prevalence, it is expected to rise in some regions, with Canada anticipating an increase to 400,000 (1%) by 2030 [6].

The exact cause of IBD remains unknown, but it is believed to be a complex interplay of genetic, environmental, and immunological factors [7]. Some theories include chronic inflammation in response to a malfunctioning immune response to commensal bacteria, autoimmune response to a luminal or mucosal antigen, or infection with a pathogenic organism that remains chronically in the gastrointestinal tissues [8,9].

**Figure 1:**
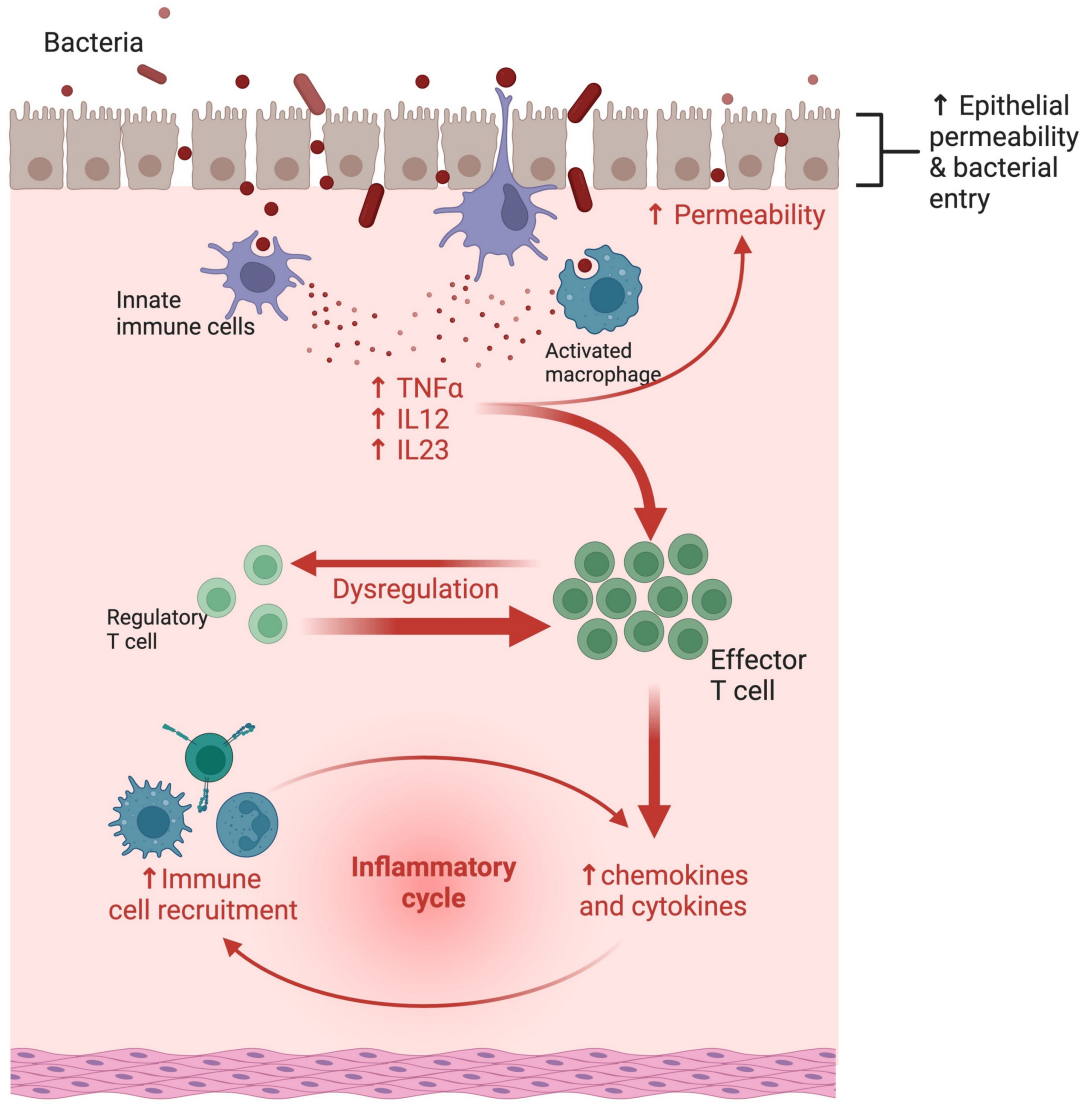
Immunopathogenesis of inflammatory bowel disease Figure adapted from Kong et al. [10] TNF-alpha: tumor necrosis factor-alpha; IL: interleukin

In IBD, the gastrointestinal immune response is disrupted due to mucosal barrier impairment as well as dysfunctional innate and acquired immune responses (Figure 1). This, in turn, causes defective secretion of pro-inflammatory cytokines such as tumor necrosis factor-alpha (TNF-alpha), IL12, and IL23 by macrophages and other immune cells, which further increases the permeability of the epithelial cells and suspects the mucosa to bacterial entry, as shown in figure 1 below. Additionally, dysfunctional T cells infiltrate the mucosa, which secretes cytokines that promote the activation and accumulation of neutrophils at the site of inflammation [7].

Physical activity, recognized for its numerous health benefits, has emerged as a potential therapeutic approach for managing chronic diseases such as IBD. However, its effectiveness as a treatment option is not yet fully understood. Regular exercise improves cardiovascular health, metabolic function, and mental well-being [11]. Recent studies suggest it may also play a role in modulating inflammatory responses and maintaining gut health, potentially influencing the course of IBD [7].

This systematic review aims to evaluate the impact of exercise on the management of IBD in adults by synthesizing evidence on its effects on disease progression, symptom relief, and overall quality of life. This review seeks to highlight the efficacy of various exercise modalities, such as aerobic exercise, resistance training, and mind-body interventions, as therapeutic interventions for IBD. The findings aim to inform clinical practice by advocating for personalized exercise programs tailored to the unique needs of IBD patients, ultimately enhancing their management strategies and improving patient outcomes.

## Review

Methods

Search Strategy and Selection Criteria

A systematic literature search was conducted between May 12 and May 22, 2024, to investigate the effects of physical exercise on the disease progression of IBD. The following electronic databases were searched: PubMed/MEDLINE, Cochrane Library, and Google Scholar. Additionally, clinical trial registries, such as ClinicalTrials.gov and the International Standard Randomised Controlled Trial Number (ISRCTN) registry, were searched to identify completed and published studies. The search process followed the PRISMA 2020 guidelines.

The search strategy utilized a combination of relevant keywords, Medical Subject Headings (MeSH) terms, and Boolean operators (AND, OR, NOT) related to IBD, exercise, and disease activity (Table 1).

**Table 1:** Search strategy ISRCTN: International Standard Randomised Controlled Trial Number

Database Used	Search Strategy
PubMed/MEDLINE	("disease progression" OR progression* OR progress* OR "clinical course" OR "clinical progression" OR "exacerbation disease" OR "disease exacerbat*" OR "progression disease" OR symptom* OR exaggerat* OR magnifi* OR increas* OR worsen* OR exacerbat* OR flare* OR "flare up" OR flareup* OR (flared OR flaring) AND up) AND ("inflammatory bowel disease*" OR "Crohn Disease" OR "colitis, ulcerative" OR "bowel disease inflammatory" OR "Inflammatory Bowel Disease 1" OR "Ulcerative Colitis Type" OR proctocolitis OR "Colitis Gravis" OR "colitis granulomatous" OR "Granulomatous Colitis" OR "Regional Ileitis" OR "Terminal Ileitis" OR "ileitis terminal" OR "ileitis regional" OR "enteritis regional" OR "Granulomatous Enteritis" OR "enteritis granulomatous" OR "Crohn's Enteritis") AND (physical* OR exercise* OR activit* OR isometric)
Cochrane	MeSH descriptor: (Exercise) explode all trees OR ((physical NEXT (exercise* OR activit*))):ti,ab,kw OR ((isometric NEXT excercis*)):ti,ab,kw AND MeSH descriptor: (Disease Progression) explode all trees OR (progression* OR symptom* OR exaggeration* OR magnification* OR increase*):ti,ab,kw OR (clinical NEXT (course* OR progression*)):ti,ab,kw OR (disease NEXT (exacerbation* OR exaggeration* OR worsening)):ti,ab,kw OR (symptom*):ti,ab,kw OR ((flare-up*) OR (flare NEXT up*) OR (flaring NEXT up*) OR flare*):ti,ab,kw AND MeSH descriptor: (Inflammatory Bowel Diseases) explode all trees OR ("inflammatory NEXT bowel NEXT disease OR (1)"):ti,ab,kw OR ((ulcerative NEXT colitis) OR proctocolitis OR colitis OR (colitis NEXT gravis) OR (granulomatous colitis)):ti,ab,kw OR ((regional Ileitis) OR (terminal Ileitis) OR (regional NEXT enteritis) OR (granulomatous enteritis) OR (Crohn's enteritis) OR (regional enteritis) OR ileocolitis):ti,ab,kw
ISRCTN	Text search: physical activity or exercise; condition: inflammatory bowel disease
Google Scholar	With all of the words: exercise AND inflammatory bowel disease
ClinicalTrials.gov	Condition/disease: inflammatory bowel disease Intervention/treatment: physical activity

A comprehensive search strategy was employed to identify relevant studies. Duplicate records were removed using Mendeley's automatic deduplication function and further verified through manual screening. The search was limited to studies published in English from 2000 onwards. Review articles, meta-analyses, case reports, editorials, animal studies, and abstracts were excluded to ensure the inclusion of primary research with the most recent and relevant data.

The final search terms and their combinations used in PubMed and other databases were documented for transparency and potential replication. Following the initial screening of titles and abstracts, potentially eligible studies were retrieved and assessed in full text to determine their final inclusion based on the pre-defined criteria (Table 2).

**Table 2:** Inclusion and exclusion criteria RCT: randomized controlled trial; CCT: controlled clinical trial; IBD: inflammatory bowel disease; CDAI: Crohn's disease activity index

	Inclusion Criteria	Exclusion Criteria
Date	Studies conducted after the year 2000	Studies conducted before the year 2000
Language	English studies	Studies published in any language other than English
Study design	RCTs, non-randomized control trials, CCTs with clear randomization, or control group allocation methods. Observational studies (cohort studies and case-control studies) with well-defined methodologies and appropriate control for confounding factors	Reviews, editorials, case reports, abstracts, and animal studies
Participants	Adults 18+ diagnosed with IBD confirmed by colonoscopy and/or biopsy. Clearly defined criteria for participant selection and disease classification	Studies involving participants under the age of 18, with additional severe co-morbidities that could significantly impact results (e.g., uncontrolled heart disease, recent major surgery)
Additional criteria	Studies reporting on at least one of the following outcomes related to IBD progression: Changes in symptoms (e.g., abdominal pain, diarrhea, rectal bleeding) using validated scoring systems (specify the systems if applicable). Hospitalization rates for IBD-related complications. Need for surgery related to IBD. Changes in disease activity scores (specify the scoring systems to be used, e.g., CDAI, Mayo Score for Ulcerative Colitis)	Studies with unclear methodology or high risk of bias as assessed by a standardized tool

Systematic Search and Critical Appraisal

A comprehensive search strategy was employed to identify relevant studies on the impact of physical activity in adult patients with IBD. This ensured a thorough evaluation of all available evidence for deriving robust conclusions on the effectiveness of exercise interventions.

Two independent reviewers (G.S. and H.A.) extracted data from the selected articles using the "checklist for critical appraisal and data extraction for systematic reviews of prediction modeling studies" [12]. The extracted data encompassed study design, participant characteristics, predictors, outcomes, model development methods, performance metrics, validation statistics, and potential clinical applications.

To assess the methodological quality and potential bias within the selected studies, we employed established tools tailored to the specific study design. Randomized controlled trials (RCTs) were evaluated using the Cochrane risk-of-bias 2 tool for randomized trials (RoB 2) [13]. For cohort studies, we utilized the Newcastle-Ottawa Scale (NOS) [14]. Finally, the Joanna Briggs Institute (JBI) critical appraisal checklist for quasi-experimental studies was used to assess non-randomized experimental studies [15].

Discrepancies in assessment were resolved through a consensus meeting with the senior author (I.M.), ensuring the reliability and robustness of the review findings for future research.

Results

Search Results

**Figure 2:**
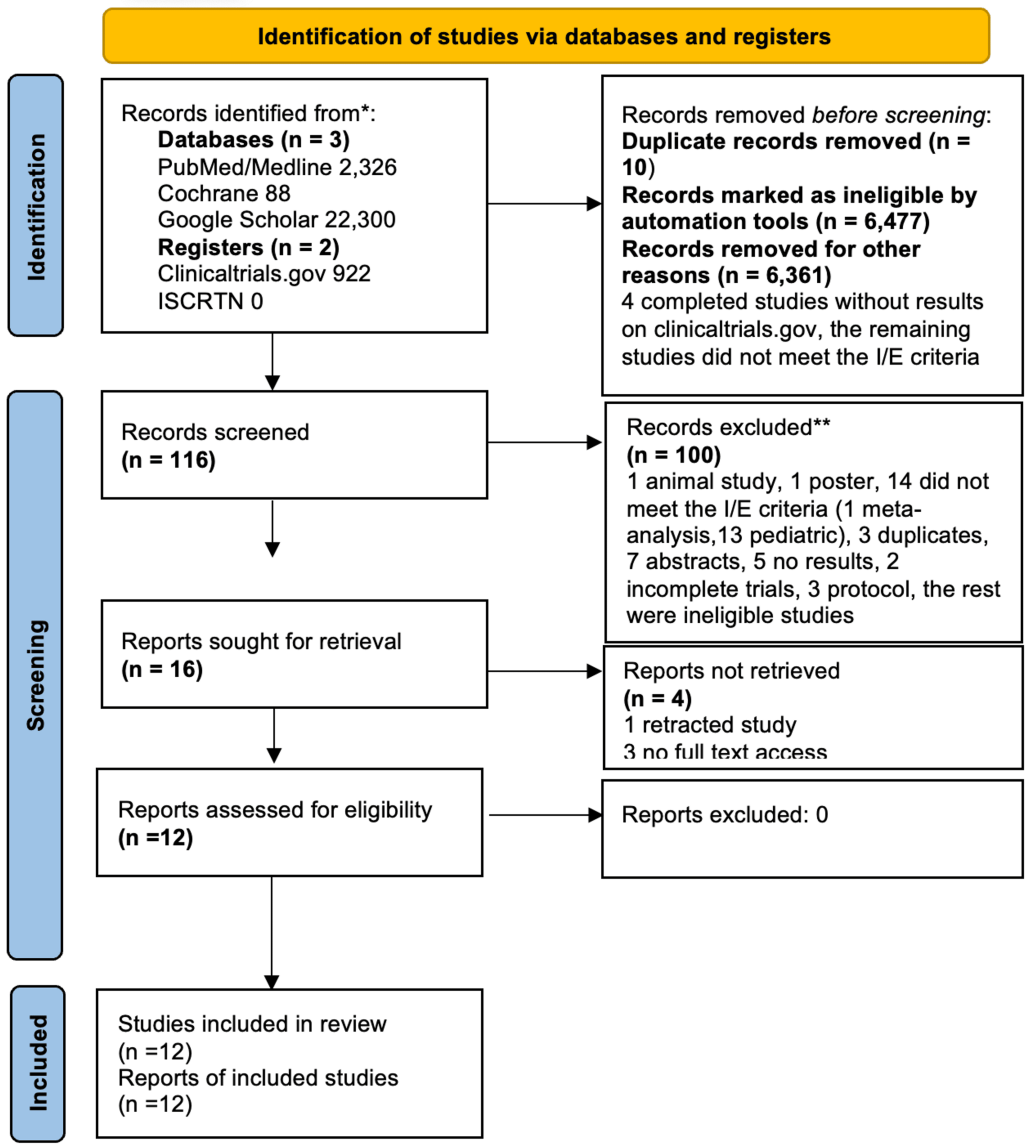
PRISMA flow diagram PRISMA: Preferred Reporting Items for Systematic Reviews and Meta-Analyses

A comprehensive search strategy identified a total of 2,326 studies in PubMed/MEDLINE, 88 in Cochrane, 22,300 in Google Scholar, 922 on ClinicalTrials.gov registries, and none on ISRCTN. Applying the inclusion and exclusion criteria outlined in Table 2 resulted in the exclusion of 6,477 studies. After removing duplicates using both Mendeley's automatic function and manual duplicate removal, the remaining 116 studies were screened according to the PRISMA flow diagram (Figure 2), which ultimately included 12 studies.

Risk-of-Bias Assessment

The risk of bias within the included studies (n=12) was assessed using tools appropriate for each study design (e.g., Cochrane's RoB 2 tool for RCTs, NOS for cohort studies, and JBI critical appraisal tool for non-randomized interventional studies) (Table 3). The risk of bias varied across studies. Most RCTs were assessed as having a low risk of bias, while a few had some concerns related to incomplete outcome data, allocation concealment, or potential for detection or unmeasured confounding bias (Table 3). The non-randomized trials and the other cohort studies assessed by NOS and JBI, respectively, were generally considered to be of low risk of bias. Due to the absence of high-risk or low-quality studies, all studies were included in the final analysis to ensure a comprehensive understanding of the evidence. This approach prioritized the inclusion of relevant data while acknowledging potential limitations in study quality.

**Table 3:** Characteristics and quality assessment of studies on exercise interventions in inflammatory bowel disease RCT: randomized controlled trial

Study	Design	Title	Quality Assessment Tool	Quality Assessment Results	Inclusion Decision
Tew et al., 2019 [16]	RCT	High-intensity interval training and moderate-intensity continuous training in adults with Crohn's disease: A pilot randomized controlled trial	Cochrane Risk of Bias 2 tool	Low risk of bias	Included
Ng et al., 2007 [17]	RCT	Low-intensity exercise improves quality of life in patients with Crohn's disease	Cochrane Risk of Bias 2 tool	Low risk of bias	included
Klare et al., 2015 [18]	RCT	The impact of a ten-week physical exercise program on health-related quality of life in patients with inflammatory bowel disease: a prospective randomized controlled trial	Cochrane Risk of Bias 2 tool	Low risk of bias	Included
Seeger et al., 2020 [19]	RCT	Moderate endurance and muscle training are beneficial and safe in patients with quiescent or mildly active Crohn’s disease	Cochrane Risk of Bias 2 tool	Low risk of bias	Included
Gerbarg et al., 2015 [20]	RCT	The effect of breathing, movement, and meditation on psychological and physical symptoms and inflammatory biomarkers in inflammatory bowel disease: a randomized controlled trial	Cochrane Risk of Bias 2 tool	Some concerns of bias- incomplete outcome data, allocation concealment bias	Included
Cronin et al., 2019 [21]	RCT	Moderate-intensity aerobic and resistance exercise is safe and favorably influences body composition in patients with quiescent Inflammatory Bowel Disease: a randomized controlled cross-over trial	Cochrane Risk of Bias 2 tool	Some concerns of bias- potential for detection bias	Included
Langhorst et al., 2007 [22]	RCT	Effects of a comprehensive lifestyle modification program on quality-of-life in patients with ulcerative colitis: a twelve-month follow-up	Cochrane Risk of Bias 2 tool	Low risk of bias	Included
Sigurdsson et al., 2021 [23]	Cohort	Physical exercise is associated with beneficial bone mineral density and body composition in young adults with childhood-onset inflammatory bowel disease	Newcastle–Ottawa quality assessment scale (NOS) for cohort studies	Low risk of bias	included
Patricia et al., 2015 [24]	Cohort	Exercise decreases risk of future active disease in inflammatory bowel disease patients in remission	Newcastle–Ottawa quality assessment scale (NOS) for cohort studies	Some concerns of bias- potential unmeasured confounding	Included
Lamers et al., 2021 [25]	Non-randomized control trial	Repeated prolonged moderate-intensity walking exercise does not appear to have harmful effects on inflammatory markers in patients with inflammatory bowel disease	JBI Critical Appraisal tool for non-randomized quasi-experimental studies	Low risk of bias	Included
Spijkerman et al., 2021 [26]	Non-randomized control trial	Refractory neutrophils and monocytes in patients with inflammatory bowel disease after repeated bouts of prolonged exercise	JBI Critical Appraisal tool for non-randomized quasi-experimental studies	Low risk of bias	Included
van Erp et al., 2021 [27]	Non-randomized control trial	Improvement of fatigue and quality of life in patients with quiescent inflammatory bowel disease following a personalized exercise program	JBI Critical Appraisal tool for non-randomized quasi-experimental studies	Low risk of bias	Included

Most RCTs were assessed as having a low risk of bias, while a few had some concerns related to incomplete outcome data, allocation concealment, or potential for detection or unmeasured confounding bias (Table 4).

**Table 4:** Risk of bias assessment (Cochrane RoB 2) for RCTs +: low risk of bias; !: moderate risk of bias; -: high risk of bias D1: randomization process; D2: deviations from the intended interventions; D3: missing outcome data; D4: measurement of the outcome; D5: selection of the reported result

Study	D1	D2	D3	D4	D5	Overall
Tew et al., 2019 [16]	+	+	+	+	+	+
Ng et al., 2007 [17]	+	+	+	+	+	+
Klare et al., 2015 [18]	+	+	+	+	+	+
Seeger et al., 2020 [19]	+	+	+	+	+	+
Gerbarg et al., 2015 [20]	!	+	+	+	+	!
Cronin et al., 2019 [21]	+	+	+	!	+	!
Langhorst et al., 2007 [22]	+	+	+	+	+	+

The non-randomized trials and the other cohort studies assessed by NOS and JBI, respectively, were generally considered to be of low risk of bias (Tables 5, 6). Studies assessed by both NOS and JBI tools with a minimum score of 7/9 were included. Notably, one cohort study by Patricia et al., 2015 raised concerns for potential detection or unmeasured confounding bias, which could have influenced the observed association between physical activity and disease activity [24].

**Table 5:** Newcastle-Ottawa quality assessment scale and results of cohort studies - no score awarded; * one point awarded out of nine for quality assessment; ** two points awarded out of nine for quality assessment Selection: assesses how well the study minimizes bias in choosing participants. Maximum score: four stars; Comparability: evaluates how effectively the study accounts for differences between exposed and unexposed groups at the outset. Maximum score: two stars; Outcome: examines how well the study measures the outcome of interest and minimizes bias in its ascertainment. Maximum score: three stars; Total maximum score: nine stars

Author (Year)		Sigurdsson et al., (2021) [23]	Patricia et al., (2015) [24]
Study type		Cohort	Cohort
Selection	Representativeness of the exposed cohort	*	*
	Selection of the non-exposed cohort	*	*
	Ascertainment of exposure	*	-
	Demonstration that outcome of interest was not present at the start of the study	*	-
Comparability	Comparability of cohorts on the basis of the design or analysis	**	**
Outcome	Assessment of outcome	-	-
	Was follow-up long enough for outcomes to occur	*	*
	Adequacy of follow-up of cohorts	*	**
Final score		8/9	7/9

**Table 6:** JBI quality assessment of included non-randomized experimental studies Total maximum score: 9 JBI: Joanna Briggs Institute

JBI Critical Appraisal Checklist for Quasi-Experimental Studies	Lamers et al., 2021 [25]	Spijkerman et al., 2021 [26]	Sigurdsson et al., 2021 [23]
Is it clear in the study what is the ‘cause’ and what is the ‘effect’ (i.e., there is no confusion about which variable comes first)?	yes	yes	yes
Were the participants included in any comparisons similar?	yes	yes	yes
Were the participants included in any comparisons receiving similar treatment/care, other than the exposure or intervention of interest?	yes	yes	yes
Was there a control group?	yes	yes	yes
5. Were there multiple measurements of the outcome both pre- and post-intervention/exposure?	yes	yes	yes
6. Was follow-up complete, and if not, were differences between groups in terms of their follow-up adequately described and analyzed?	yes	yes	yes
7. Were the outcomes of participants included in any comparisons measured in the same way?	yes	yes	yes
8. Were outcomes measured in a reliable way?	yes	yes	yes
9. Was appropriate statistical analysis used?	yes	yes	yes
Final score	9/9	9/9	9/9

Due to the absence of high-risk or low-quality studies, all studies were included in the final analysis to ensure a comprehensive understanding of the evidence. This approach prioritized the inclusion of relevant data while acknowledging potential limitations in study quality. By employing a rigorous quality assessment process using established tools appropriate for each study design (e.g., RoB 2 tool for RCTs, NOS for cohort studies), the review ensured that the synthesized findings were derived from studies with an adequate level of internal validity. This methodological rigor strengthens the overall reliability and trustworthiness of the conclusions drawn from this review.

Study Characteristics

The 12 included studies employed a variety of designs, with seven being RCTs, three being non-RCTs, and two being observational cohort studies (Table 3). Sample sizes ranged from 19 to 921 participants. Treatment duration varied from three days to six months, with follow-up periods also differing across studies. The interventions and outcomes investigated varied as well. However, the majority of studies focused on the relationship between physical activity (aerobic, resistance training, or other methods) and standardized measures of disease activity. These measures often included questionnaires and objective assessments of inflammation (Table 7).

**Table 7:** Summary of studies investigating the effects of exercise on IBD RCT: randomized controlled trial; CD: Crohn's disease; UC: ulcerative colitis; IBD: inflammatory bowel disease (includes CD and UC); MICT: moderate-intensity continuous training; HIIT: high-intensity interval training; PRO-2: patient-reported outcome score 2; CDAI: Crohn's disease activity index; IBDQ: inflammatory bowel disease questionnaire; sIBDQ: short inflammatory bowel disease questionnaire; BBMW: breath-body-mind workshop; CBC: complete blood count; CRP: C-reactive protein (inflammatory marker); BMI: body mass index; VO2 max: maximum volume of oxygen (cardiorespiratory fitness measure); DEXA scan: dual-energy X-ray absorptiometry, (bone density scan); BSI-18: brief symptom inventory (mental health screening); BAI: Beck anxiety inventory; BDI: Beck depression inventory; PDS: perceived disability scale; PSQ: perceived stress questionnaire; SF-36: short-form 36 health survey (quality of life questionnaire); CAI: colitis activity index; fMLF: N-formylmethionine-leucyl-phenylalanine (bacterial/mitochondrial stimulant); CPET: cardiopulmonary exercise testing; HRQoL: health-related quality of life; CIS: Chalder fatigue scale

Author, Year	Study Design	Population	Control Group	Intervention	Treatment Duration/Follow-up	Outcomes Measured	Key Results
Tew et al., 2019 [16]	RCT	45 patients between 16 and 65 years of age with a clinical diagnosis of CD	Usual care	MICT and high-intensity HIIT exercise three sessions a week	12 weeks	Body composition (mass, stature, waist circumference) Blood pressure & resting heart rate Cardio fitness (ventilatory threshold, peak oxygen uptake) Disease status (CDAI) & intestinal inflammation (fecal calprotectin) Blood markers of inflammation Standard questionnaires at 3 & 6 months	Cardio Improvement: HIIT led to a greater increase in peak oxygen uptake (2.4 vs. 0.7 mL/kg/min) compared to MICT. Patient-Reported Benefits: Both groups reported physical benefits and disease-specific improvements such as reduced inflammation, less frequent bowel movements, and improved gut feeling. Relapse: one participant from each group experienced a relapse
Ng et al., 2007 [17]	RCT	32 adults with mildly active Crohn’s disease or in remission	Usual care	Three sessions of walking each week	Three months	International Physical Activity Long Questionnaire, Harvey-Bradshaw Simple Index of Crohn's disease, the IBDQ, and IBD stress index	IBD Stress Test & IBDQ: Significant improvement (p < 0.05) for the exercise group, no significant change for control. Harvey-Bradshaw Index: Significant change (p < 0.05) in both groups, but the exercise group showed improvement (p < 0.01) while the control worsened (p = 0.04)
Klare et al., 2015 [18]	RCT	132 patients who are 18 years or older with either CD or UC diagnosed at least one year before screening, patients in remission or with mild active disease	Usual care	Running program (adapted for baseline BMI) for 10 weeks, 3x/week Supervised outdoor running at moderate intensity (increased heart rate, able to talk while running)	10 weeks	Primary: change in total IBDQ score (quality of life) after 10 weeks. Secondary: Changes in disease activity scores (CDAI, RI), Body weight, Inflammation markers (white blood cell count, CRP, fecal calprotectin)	IBDQ significantly improved in all dimensions (emotional, bowel, social, systemic) after the program (p<0.004). Control Group: IBDQ total score improved (p=0.004). Significant improvements in emotional, bowel, and systemic dimensions (p<0.021). Social dimension score did not improve significantly (p=0.151)
Seeger et al., 2020 [19]	RCT	45 CD patients aged between 18 and 65 years and diagnosed with CD for at least six months, quiescent or mildly active disease, and stable medication for at least four weeks	Usual care (no exercise intervention)	Moderate Endurance: 30 min, three times a week (preferred activity) 60-80% max heart rate (220-age) Moderate intensity using Borg scale (able to talk) Muscle Strength: 30-40 min, three times per week 12 bodyweight exercises	12 weeks	Baseline and three months: vital Signs (blood pressure, pulse, and respiratory rate), Anthropometry (height, weight, BMI), blood tests (CBC, kidney, liver, pancreas, iron, cholesterol, and triglycerides), Stool Tests (fecal calprotectin) Inflammation Markers Muscle Strength (upper & lower body) Questionnaires (sIBDQ, sIPAQ, PRO-2, CDAI)	CDAI: muscle group: improved (decreased) from 95 to 88. Endurance group: no change (remained around 84). Control group: worsened (increased) from 64 to 73. Quality of Life (sIBDQ): improved in all exercise groups (endurance, muscle) but only statistically significant for emotional function in the endurance group after three months. Control group likely saw a decrease in quality of life (not explicitly stated). Muscle Strength: increased in both endurance and muscle groups. Control group: significant decrease in strength
Gerbarg et al., 2015 [20]	RCT	29 patients with IBD (aged 18–85 years)	Educational seminar group: included one six-hour day followed by two 90-minute sessions (total of nine hours) during which they received information about IBD and its treatment	The nine-hour BBMW workshop focused on stress management and relaxation techniques, delivered over two days	26 weeks	Time Points: 0 weeks (baseline), 6 weeks, 26 weeks. Psychological Measures: BSI-18 (general mental health), BAI (anxiety), BDI (depression), IBD Symptoms & Quality of Life: IBDQ, PDS (psycho-social distress), PSQ (perceived stress). Physiological Measures: body temperature, blood pressure pulse, inflammatory biomarkers like fecal calprotectin (stool test) and CRP (blood test)	BBMW group: significant improvements (compared to baseline) at week six: BSI-18, BAI, IBDQ. Significant improvements (compared to baseline) at week 26: BSI-18, BAI, BDI, IBDQ, PDS, PSQ, and decreased CRP. Educational seminar group: no significant changes in any measures at week six or 26
Cronin et al., 2019 [21]	RCT	110 patients, aged 18 to 40 years with a diagnosis of IBD in disease remission and with a BMI of 22 to 35 kg/m2, who were physically inactive or had low levels of activity with no involvement in regular or organized exercise in the month prior to recruitment	Maintained usual levels of physical activity (none or low)	Combined aerobic and resistance training programs of moderate intensity based on the couch to five-kilometer training programs	Eight weeks	Primary Outcome: change in body composition measured by DEXA scan. Secondary outcomes: disease activity scores (specific scales for CD and UC), quality of life measures, anxiety and depression levels, blood levels of inflammatory markers, and changes in gut microbiome diversity	Improved fitness: The exercise group showed increased VO2 max (a measure of cardiorespiratory fitness) compared to the control. Better body composition: The exercise group had reduced body fat and increased lean muscle mass compared to control. No worsening of disease activity: exercise did not worsen IBD activity scores. Gut microbiome: No significant changes were observed in the gut microbiome diversity
Langhorst et al., 2007 [22]	RCT	60 patients with UC in clinical remission or with low disease activity were randomized	Usual care	Ten-week program (60 hours total) focused on stress management, training, learning about IBD (psycho-educational elements), developing self-care strategies	Ten weeks	Quality of life: using the IBDQ Psychological distress: using the BSI Physical function: using a part of the SF-36 questionnaire Clinical disease activity: using the CAI)	Three months after lifestyle changes: The intervention group improved significantly in physical function (SF-36, p=0.0175) and anxiety (BSI, p=0.0294). Relaxation techniques predicted better psychological well-being (p=0.034). No significant improvements in IBD symptoms (IBDQ), disease activity, or hospitalizations. No significant changes at 12 months
Lamers et al., 2021 [25]	Non-randomized control trial	19 IBD patients	Two control groups: non-IBD walkers: test effect of disease (healthy participants who walked) IBD non-walkers: test effect of exercise (IBD patients who did not walk)	IBD walkers and non-IBD walkers walked 30, 40, or 50 km at a self-selected pace on four consecutive exercise days.	Four consecutive days	Blood: cytokine levels (inflammation markers) in both IBD and non-IBD walkers - collected at baseline and after each exercise day. Stool: calprotectin (inflammation marker) in IBD walkers and non-walkers - collected at baseline, days 2-3, and after the exercise event. Questionnaires: disease activity in IBD walkers - measured at baseline and after the exercise event	Cytokines: the changes in cytokine concentrations (IL-6, IL-8, IL-10, IL-1β, TNF-α) were overall similar between IBD walkers and non-IBD walkers. However, there was a temporary significant increase in IL-6 (p < .001) and IL-10 (p = .006) from baseline to day 1 after exercise. Fecal calprotectin: exercise did not significantly affect fecal calprotectin levels (p = .48). Clinical disease activity: UC: no significant change (p = .92). CD: slight increase (p = .024), requiring further investigation
Spijkerman et al., 2021 [26]	Non-randomized control trial	19 patients with IBD age ≥ 18 years and diagnosis of CD or UC not using specific biologicals (infliximab, adalimumab, golimumab, ustekinumab)	19 healthy patients completed a distance of 30- 50 km/day on three consecutive days at a self-selected pace	IBD patients four-day walking event (30-50km/day)	Four-day walking event	Responsiveness to the bacterial/mitochondrial-stimulus fMLF was tested in granulocytes and monocytes blood cells	Increased immune response in all, but IBD patients showed lower response
van Erp et al., 2021 [27]	Non-randomized control trial	25 IBD patients aged between 18 and 60 years with a diagnosis of IBD including CD, UC, and IBD-unclassified	-	12-week personalized exercise program	12 weeks	Fatigue (CIS), HRQoL (IBDQ), cardiorespiratory fitness (CPET)	Fatigue significantly improved (p < 0.001) HRQoL significantly improved (p < 0.001) Improved maximum power output (p=0.002)
Sigurdsson et al., 2021 [23]	Cohort	114 patients with childhood-onset IBD	Three population-based control cohorts from two urban regions in the southwest of Sweden were pooled and used as controls for the measurements of physical exercise habits and body composition	A standardized physical exercise questionnaire to gather information about present physical exercise patterns in the last 12 months	-	Bone mineral density (DEXA scan) and body composition	Less exercise in IBD patients (57% vs 68% of controls, p=0.053). Inactive IBD patients had lower bone density and muscle mass (all p<0.05). Highly active IBD patients had similar bone density and muscle mass as controls except for the spine and hip (p=0.007 & 0.015, respectively)
Patricia et al., 2015 [24]	Cohort	Adult patients (greater than or equal to 18 years of age) with self-reported IBD or indeterminate colitis (IC) who were in remission	-	Exercise assessed using the Godin Leisure-Time Activity Index. Self-reported physical activity levels were measured	Six months	The primary outcome of interest was the presence of active disease at 6 months on the follow-up survey	Higher exercise linked to lower risk of disease activity flare-up at 6 months for both: CD: adjusted risk ratios (RR)=0.72, 95% confidence interval (CI)=0.55-0.94 UC: adjusted RR=0.78, 95% CI=0.54-1.13)

Discussion

IBD is a persistent inflammatory bowel disorder that affects the gastrointestinal tract, with UC and CD being its two most prevalent forms. These conditions considerably diminish the quality of life of millions globally, manifesting symptoms such as abdominal pain, diarrhea, fatigue, and weight loss. Furthermore, up to 60% of IBD patients suffer from extra-intestinal symptoms, particularly musculoskeletal issues such as osteoporosis or muscle degeneration, further increasing their overall health burden [2]. Despite recent advancements in the treatment of IBD using novel biological and immunomodulating therapies, many IBD patients do not achieve remission and often suffer from numerous adverse effects of long-term medication. Hence, many patients resort to alternative medicine and lifestyle changes to help achieve remission. Physical exercise has been suspected to affect the progression of inflammation in several idiopathic autoimmune diseases. The studies reviewed provided valuable insights into the potential benefits of exercise and physical activity for individuals suffering from IBD.

The RCTs consistently demonstrated positive effects of exercise interventions. Tew et al. (2019) found that high-intensity interval training (HIIT) resulted in greater increases in cardiorespiratory fitness compared to moderate-intensity continuous training (MICT) in CD patients [16]. Both exercise groups reported physical and disease-specific benefits, such as reduced inflammation and improved gut feeling. Ng et al. (2007) showed that a three-month walking program led to significant improvements in IBD-related stress and quality of life for the exercise group, while the control group experienced worsening of disease activity [17].

Klare et al. (2015) reported significant improvements in quality of life across all dimensions (emotional, bowel, social, systemic) after a 10-week running program in CD and UC patients [18]. Seeger et al. (2020) found that both moderate endurance and muscle strength training programs led to improvements in Crohn's disease activity, quality of life, and muscle strength, while the control group saw a worsening of disease activity [19]. Gerbarg et al. (2015) demonstrated that a mind-body intervention focused on stress management and relaxation techniques resulted in improvements in mental health, quality of life, and inflammatory markers in IBD patients, while the control group showed no significant changes [20].

Cronin et al. (2019) showed that an eight-week combined aerobic and resistance training program improved fitness and body composition without worsening disease activity in IBD patients [21]. Langhorst et al. (2007) found that a 10-week lifestyle intervention program led to improvements in physical function and anxiety but no significant changes in IBD symptoms or disease activity [22].

The non-randomized studies provided further evidence for the benefits of physical activity in IBD. Lamers et al. (2021) found that a four-day walking event did not significantly affect fecal calprotectin levels or disease activity in IBD patients despite temporary increases in some inflammatory cytokines [25]. Spijkerman et al. (2021) reported that IBD patients showed a lower immune response to the bacterial/mitochondrial stimulus than healthy controls during the same walking event [26].

The cohort studies also offered relevant insights. Sigurdsson et al. (2021) demonstrated that inactive IBD patients had lower bone density and muscle mass than controls, while highly active IBD patients had similar measures to the control group [23]. Patricia et al. (2015) found that higher self-reported physical activity levels were associated with a lower risk of disease activity flare-ups in both CD and UC patients over a six-month period [24].

Overall, the studies reviewed suggest that exercise and physical activity can have a positive impact on various outcomes for IBD patients, including cardiorespiratory fitness, quality of life, disease activity, and inflammation. The studies employed a range of exercise modalities, including aerobic, resistance, and mind-body interventions, indicating that multiple types of physical activity may be beneficial. However, the studies also highlight the need for further research to better understand the optimal exercise prescription and the potential mechanisms underlying the observed benefits.

The findings from the studies reviewed in the present discussion are largely consistent with the conclusions of two recent systematic reviews on the role of physical activity in the prevention and management of IBD. Protano et al. (2024) conducted a systematic review that assessed the potential role of physical activity in reducing the risk of developing IBD and in the management of the disease [28]. They found that four out of six studies investigating the relationship between physical activity and IBD risk showed an inverse relationship, suggesting that physical activity may have a protective effect against the development of IBD. Additionally, the review reported positive effects of physical activity on IBD symptoms, as well as improvements in comorbidities, complications, and quality of life. Raman et al. (2022) provided a narrative review on physical activity in patients with IBD, discussing the effects of physical activity on disease onset, disease course, and important patient-reported outcome measures (PROMs) [29]. The review highlighted the potential benefits of physical activity in maintaining clinical remission and improving quality of life, fatigue, depression, and anxiety in IBD patients. However, the authors cautioned that further research is needed to establish specific exercise recommendations for IBD patients based on disease severity and individual characteristics.

These systematic reviews collectively support the notion that physical activity plays a significant role in the prevention and management of IBD, with potential benefits on disease activity, quality of life, and mental health outcomes.

Strengths of the review

This systematic review stands out for its robust methodology and comprehensive approach to understanding the effects of physical exercise on IBD management. The search strategy effectively minimized publication bias by encompassing studies from multiple databases, ensuring a broad look at the relevant research. This is particularly important given the acknowledged variability in exercise programs and IBD presentations. By highlighting the need for future research to target specific populations and interventions, the review emphasizes the importance of tailoring exercise recommendations for optimal effectiveness.

The review's strength lies in its diverse inclusion of study designs. It incorporated the gold standard of RCTs alongside observational studies and cohort analyses. This multifaceted approach provides a rich tapestry of evidence, offering a comprehensive understanding of how physical exercise impacts IBD. Furthermore, the review prioritized high-quality RCTs, minimizing bias and strengthening the overall conclusions drawn from the evidence.

To ensure the findings remained relevant to current practices, the review specifically focused on studies conducted in the 21st century. This contemporary focus guarantees the applicability of the review's conclusions to present-day contexts. Moreover, the review team meticulously evaluated the methodological quality of each included study using established tools. This rigorous quality assessment process, employing tools such as the NOS and the Cochrane RoB 2 tool, bolstered the reliability of the review's conclusions by upholding strict standards for internal validity within the analyzed studies.

Finally, the review's strength lies in its ability to draw upon studies from diverse sources and locations. By consolidating evidence from a broad range of populations and settings, the review enhances the generalizability of its conclusions. This broader applicability makes the review's results more valuable for informing clinical decision-making and guiding actual practices in IBD management. In conclusion, these combined strengths contribute significantly to the credibility, relevance, and overall reliability of the systematic review, offering valuable insights into the potential benefits of physical exercise as a therapeutic approach for managing IBD.

Limitations of the review and included studies

While this systematic review offers valuable insights, interpreting its findings requires considering some limitations. The included studies varied in quality due to differences in design, sample size, and outcome measures, potentially impacting the overall strength of the evidence.

Another concern lies in blinding. In some studies, participants or assessors were aware of the intervention (exercise vs. control), which, while understandable for exercise interventions, could introduce bias into the results. Additionally, selective outcome reporting in one study and small sample sizes with heterogeneity in other studies raise questions about the generalizability of the findings. Furthermore, a lack of detailed information on exposure and outcome ascertainment in another study introduces uncertainty. Finally, the language restriction potentially excluded relevant non-English research, limiting comprehensiveness and introducing potential bias.

Future directions for research on physical activity and IBD management

Despite the valuable insights gleaned from this systematic review, several key areas warrant further exploration to solidify the understanding of physical activity's role in managing IBD.

One crucial future direction lies in conducting longitudinal studies with extended follow-up periods. These studies would enable researchers to assess the sustained impact of various exercise interventions on disease progression, symptom management, and overall quality of life among adult IBD patients. This information is vital for determining the long-term effectiveness of exercise as a therapeutic approach.

Another gap in current knowledge concerns the influence of psychosocial factors on the relationship between physical activity and IBD outcomes. Future research should investigate how stress, mental health status, and social support systems interact with exercise to influence disease management in individuals with IBD. Understanding these interrelationships can help tailor interventions for optimal benefit.

Furthermore, comparative effectiveness research is needed to determine the most efficacious exercise modalities for the IBD population. Studies comparing the effectiveness of aerobic exercise, resistance training, yoga, and other potential interventions can help guide clinicians in recommending the most suitable exercise programs based on individual needs and preferences.

The development of personalized exercise prescriptions represents another promising future direction. By tailoring exercise programs to consider individual disease characteristics, patient preferences, and any existing comorbidities, researchers can optimize interventions for improved adherence and maximize benefits for patients with IBD.

By addressing these research gaps and pursuing these identified future directions, we can significantly advance our understanding of how physical activity can contribute to effective IBD management. This will ultimately lead to improved outcomes and a better quality of life for individuals living with this chronic condition.

## Conclusions

This systematic review thoroughly evaluated the impact of physical activity on adults with IBD, employing a comprehensive search strategy that yielded high-quality studies and robust findings. It underscores the necessity for personalized exercise prescriptions and comparative effectiveness research on various modalities, including aerobic exercise, resistance training, and yoga, to identify the most effective approaches for alleviating symptoms and enhancing disease-related outcomes. Additionally, the review highlights the importance of investigating psychosocial factors that may influence the relationship between physical activity and IBD outcomes, as well as the need for long-term follow-up studies. By addressing these gaps, the review contributes to the development of tailored, evidence-based exercise interventions aimed at optimizing patient outcomes and improving holistic IBD management, thereby serving as a foundation for future research in this area.
